# The *miRNA-608* rs4919510 G>C polymorphism confers reduce coronary injury of Kawasaki disease in a Southern Chinese population

**DOI:** 10.1042/BSR20181660

**Published:** 2019-05-17

**Authors:** Yanfei Wang, Zhaoliang Lu, Lanyan Fu, Yaqian Tan, Di Che, Ping Huang, Lei Pi, Yufen Xu, Qihua Liang, Li Zhang, Xiantao Qiu, Xiaoqiong Gu

**Affiliations:** 1 Department of Cardiology, Guangzhou Women and Children’s Medical Center, Guangzhou Medical University, Guangzhou 510623, Guangdong, China; 2 Department of Clinical Biological Resource Bank, Guangzhou Institute of Pediatrics, Guangzhou Women and Children’s Medical Center, Guangzhou Medical University, Guangzhou 510623, Guangdong, China; 3 Department of Clinical Lab, Guangzhou Institute of Pediatrics, Guangzhou Women and Children’s Medical Center, Guangzhou Medical University, Guangzhou 510623, Guangdong, China

**Keywords:** coronary artery lesion, Kawasaki disease, miRNA-608

## Abstract

Kawasaki disease (KD) is also called mucocutaneous lymph node syndrome and is an acute febrile pediatric disease characterized by systemic vasculitis. KD typically occurs in children 5 years old or younger and occurs more often in males than in females. miRNA-608 has been reported to interact with interleukin-6 and affect innate immunity. The immune-mediated inflammation could induce the occurrence of KD; however, there is no previous research focused on the relationship between miRNA-608 polymorphism and the KD risk. The present study explored the correlation between the *miRNA-608* rs4919510 G>C polymorphism and the risk for KD. We recruited 532 patients with KD and 623 controls to genotype the *miRNA-608* rs4919510 G>C polymorphism with a TaqMan allelic discrimination assay. Single-locus analysis showed no significant association between miRNA rs4919510 G>C polymorphism and KD susceptibility. However in an analysis stratified by age, gender, and coronary artery lesion (CAL), we found a relationship between the *miRNA-608* rs4919510 G>C polymorphism and KD susceptibility. When KD patients were stratified by coronary injury, the CG/CC genotypes of the *miRNA-608* rs4919510 G>C polymorphism contributed to a higher occurrence of KD than that was found in the GG genotype patients (adjusted odds ratio = 0.74, 95% CI = 0.56–0.98, *P* = 0.033). The present study demonstrated that the *miRNA-608* rs4919510 G>C polymorphism may have a CAL-related relationship with KD susceptibility that has not been previously revealed.

## Introduction

Kawasaki disease (KD) is pediatric systemic vasculitis with the well-known complication of coronary artery aneurysm (CAA), which occurs in infants under the age of 5 [1]. Since the first case was reported by Tomisaku Kawasaki in Japan in 1967, there have been increasingly more cases reported in various countries. KD has now replaced rheumatic fever as the main cause of acquired heart disease in children, which caused the widespread attention of pediatric clinicians [2]. Although KD has replaced rheumatic fever and has become the most common form of acquired heart disease in children, the cause of KD remains unknown [3]. A large number of epidemiological and clinical observations indicated that KD may be caused by infectious factors. KD has the characteristics of a regional epidemic and self-limiting disease, with seasonality and high risk in infants and young children. These characteristics have prompted us to consider that the KD pathogen is a microorganism commonly prevalent in the natural environment and that it can cause asymptomatic infection in most individuals and acquired immunity in adulthood [4]. However, there is also compelling laboratory evidence of autoimmune responses directed against self-epitopes shared by the mycobacterial HSP65 and its human homolog HSP63 [5]. Most previous genome-wide association screenings have indicated that host genetic variants play important roles in the susceptibility to KD, such as *ITPKC* [6], *CASP3* [7], *ITPR3* [8], *GRIN3A* [9] and *ERAP1* [10] which have been reported as susceptible single nucleotide polymorphism (SNP). miRNAs are small noncoding RNA molecules that play an important role in controlling mRNA translation. miRNAs have shown promise as diagnostic markers for many pathogenic states, including certain cardiovascular diseases [11] and they are associated with many biological processes, including the immune response [12].

Rowley et al. [13] has reported 26 miRNAs that were significantly up-regulated in KD coronary arteries with a fold change>1.5 and a *P* value<0.05. A study showed that miR-200c and miR-371-5P were significantly up-regulated in the KD group compared with those in the control group [14]. The molecules of miRNA-608 are encoded by the miRNA-608 (hsa-miRNA-608) gene, which is located within the intron region of its host gene SEMA4G [15,16]. Hanin [17] found that miRNA-608 could interact with the validated cell division control protein 42 homolog and interleukin-6 targets *in vitro* and *in vivo* and that the consequences of these interactions can change cortisol levels and blood pressure.Yang found that esophageal squamous cell carcinoma patients with the GG genotype of *miRNA608*:rs4919510 had a 4.56-fold increased risk of high IL-6 expression compared with that in patients with the CC genotype [18]. Other studies have demonstrated that rs4919510, an SNP of miRNA-608 with the CC genotype, was associated with a worse prognosis in locally advanced rectal cancer patients treated with chemotherapy alone than that seen in the CC/CG genotype patients [19]. In colorectal cancer patients with stage III disease, *miRNA-608* rs4919510 was associated with an increased risk for recurrence and death [20]. Yi et al. found that overproduced IL-6 could participate in inhibiting peripheral blood lymphocyte apoptosis in KD patients [21]. Tan [22] used the enzyme-linked immunosorbent assay and found that KD patients express more IL-6. However, the relationships between the miRNA SNPs and KD susceptibility have rarely been reported. In the present paper, we focused on whether the *miRNA-608* rs4919510 G>C polymorphism was related to the risk of KD.

## Materials and methods

### Ethics statement

The present study was approved by the Guangzhou Women and Children Medical Center Ethics Committee (2014073009) and was conducted according to the International Ethical Guidelines for Research Involving Human Subjects stated in the Declaration of Helsinki. The children’s families provided written informed consent.

### Study population

The subjects included 532 patients with KD and 623 controls whose data were collected from January 2012 to January 2017 in the Guangzhou Women and Children Medical Center in China. The KD patients were diagnosed mainly based on the Japanese Kawasaki Disease Research Association’s comprehensive clinical diagnostic criteria [23]. The KD patients attended our hospital as outpatients with follow-ups and inpatients, and the healthy controls were children who came to our hospital for health examinations within the same time period and had no fever or other diseases. The present study was approved by the Guangzhou Women and Children Medical Center Ethics Committee (2014073009), and the children and their families provided written informed consent.

### DNA extraction and genotyping

Samples of 200 μl of anticoagulant-containing blood were collected according to the instructions of the Genomic DNA Extraction Kit. The specific procedures can be found in the literature [24–26]. The centrifuge tube was collected and quantified using a nucleic acid quantifier, which was eventually stored at <80°C. The *miRNA-608* rs4919510 G>C polymorphism was genotyped with the TaqMan reagent. The allele-specific probes were purchased from Applied Biosystems. The PCR reaction was performed in 384-well plates that were run on an ABI-Q6 Sequence Detection System machine [27]. Moreover, to ensure the quality and accuracy of the genotyping results, we randomly selected 10% of the samples for a repeat analysis, and the results were 100% concordant.

### Statistical analysis

First, we examined the Hardy–Weinberg equilibrium (HWE) of the samples. Next, the χ^2^ test was employed to assess the significant differences between cases and controls in the frequency distributions and genotypes. Odds ratios (ORs) and 95% confidence intervals (CIs) were used to quantify the association between the *miRNA-608* rs4919510 G>C polymorphism and KD susceptibility with adjustments for age and gender. The association between the *miRNA-608* rs4919510 G>C polymorphism and KD was assessed with analyses that were stratified by age, gender, and coronary artery lesion (CAL). The data analyses were performed with SAS software (Version 9.4; SAS Institute, Cary, NC, U.S.A.). A *P*-value <0.05 indicated a significant difference.

### Ethical approval and consent to participate

The present study has received the approval of the Institutional Committee of Guangzhou Women and Children’s Medical Center (2014073009). All patients provided written informed consent.

## Results

### Demographic characteristics

Table 1 shows the demographic characteristics of KD cases and controls. The average age of KD patients was 28.39 months (±24.68; range 1–166), and it was 28.48 months for the controls (±25.33; range 0.07–166). The distribution of age (*P* = 0.602) and gender (*P* = 0.143) was not significantly different between the KD cases and the controls, where 31.39% and 35.47% of the KD patients and controls, respectively, were female. The percentage of males was 68.61% and 64.53% in the KD patient group and control group, respectively. Among the KD cases, 9.59% had coronaryartery aneurysm and 31.58% had CAL.

**Table 1:** Frequency distribution of selected variables for cases and controls

Variables	Cases (*n* = 532)		Controls (*n* = 623)		P^*^
	No.	%	No.	%	
Age range, month	1.00–166.0		0.07–166		0.602
Mean ± SD	28.39 ± 24.68		28.48 ± 25.33		
<12	137	25.75	165	26.48	
12–60	351	65.98	397	63.72	
>60	44	8.27	61	9.79	
Gender					0.143
Female	167	31.39	221	35.47	
Male	365	68.61	402	64.53	
Coronary artery outcomes					
CAA	51	9.59			
NCAA	481	90.41			
Coronary injury					
CAL	168	31.58			
NCAL	364	68.42			

Abbreviations: CAA, coronary artery aneurysm; CAL, coronary artery lesion.

* Two-sided χ^2^ test for distributions between cases and controls.

### Association between the *miRNA-608* rs4919510 G>C polymorphism and the risk of KD

Table 2 shows the genotype distributions of *miRNA-608* rs4919510 G>C polymorphism in the KD case and control groups. The *miRNA-608* rs4919510 G>C genotype distribution analysis to assess HWE in the control group revealed an equilibrium (HWE = 0.779). However, there was no significant association between the *miRNA-608* rs4919510 G>C polymorphism and the KD susceptibility after adjustments for age and gender (CG vs. GG: adjusted OR = 0.84, 95% CI = 0.64–1.10, *P* = 0.203; CC vs. GG: adjusted OR = 0.82, 95% CI = 0.59–1.14, *P* = 0.231; dominant model: adjusted OR = 0.83, 95% CI = 0.65–1.07, *P* = 0.157; and recessive model: adjusted OR = 0.91, 95% CI = 0.69–1.21, *P* = 0.523).

**Table 2:** Genotype distributions of rs4919510 G>C polymorphism and Kawasaki disease susceptibility

Genotype	Cases (*n* = 532)	Controls (*n* = 623)	P^*^	Crude OR (95% CI)	*P*	Adjusted OR (95% CI)^†^	*P*^†^
rs4919510 (HWE = 0.774)							
GG	170 (31.95)	175 (28.09)		1.00		1.00	
CG	250 (46.99)	307 (49.28)		0.84 (0.64–1.10)	0.199	0.84 (0.64–1.10)	0.203
CC	112 (21.05)	141 (22.63)		0.82 (0.59–1.13)	0.226	0.82 (0.59–1.14)	0.231
Additive			0.355	0.90(0.76–1.06)	0.197	0.90 (0.77–1.06)	0.202
Dominant	362 (68.05)	448 (71.91)	0.153	0.83 (0.65–1.07)	0.153	0.83 (0.65–1.07)	0.157
Recessive	420 (78.95)	482 (77.37)	0.518	0.91 (0.69–1.21)	0.519	0.91 (0.69–1.21)	0.523

* χ^2^ test for genotype distributions between Kawasaki disease patients and controls.

† Adjusted for age and gender.

### Stratification analysis

KD is a disease that is related to age and gender, and the KD complication is CAL. The stratification analysis was based on age, gender, coronary artery outcomes, and coronary injury. In Table 3, we investigated the relationship between the *miRNA-608* rs4919510 G>C polymorphism and KD susceptibility. When the KD patients were stratified by coronary injury, the CG/CC genotypes of the *miRNA-608* rs4919510 G>C polymorphism contributed to a higher occurrence than that was found in the GG genotype patients (adjusted OR = 0.74, 95% CI = 0.56–0.98, *P* = 0.033).

**Table 3:** Stratification analysis for the association between miR608 rs4919510 G>C polymorphism and Kawasaki disease susceptibility

Variables	Cases/controls		Crude OR (95% CI)	*P*	Adjusted OR^*^ (95% CI)	P^*^
	GG	CG/CC				
Age, month						
<12	47/51	90/114	0.86 (0.53–1.39)	0.530	0.82 (0.50–1.34)	0.437
12–60	109/103	242/294	0.78 (0.57–1.07)	0.122	0.78 (0.57–1.08)	0.130
>60	14/21	30/40	1.13 (0.49–2.57)	0.780	1.09 (0.46–2.55)	0.849
Gender						
Females	52/61	115/160	0.84 (0.54–1.31)	0.448	0.81 (0.52–1.26)	0.349
Males	118/114	247/288	0.83 (0.61–1.13)	0.232	0.82 (0.60–1.12)	0.211
Coronary artery outcomes						
CAA	16/175	35/448	0.85 (0.46–1.58)	0.617	0.87 (0.47–1.61)	0.651
NCAA	154/175	327/448	0.83 (0.64–1.08)	0.158	0.83 (0.64–1.08)	0.157
Coronary injury						
CAL	44/175	124/448	1.10 (0.75–1.62)	0.625	1.11 (0.75–1.63)	0.609
NCAL	126/175	238/448	**0.74 (0.56–0.97)**	**0.032**	**0.74 (0.56–0.98)**	**0.033**

* Adjusted for age and gender. Statistically significant values are shown in bold (*P*<0.05).

## Discussion

KD is a common disease that occurs in 5-year-olds. KD can cause severe cardiovascular disease, such as coronary artery dilatation, coronary aneurysm, thromboembolism, severe arrhythmia, and even sudden death. In recent years, KD incidence has been increasing yearly, and it has now replaced rheumatic fever as the primary cause of acquired heart disease in children [28]. The etiology of KD is unknown, and the diagnosis depends mainly on clinical manifestations. The incidence of insensitivity to gamma globulin is high, and there are many critically ill children easy to have CAL. Delayed diagnosis and treatment can cause serious injury to children [29]. miRNAs are 20–25 nts in length and are noncoding ssRNA molecules that regulate gene expression. Increasingly more studies have shown that miRNA polymorphism could affect target genes at the posttranscriptional level by binding to the 3′-UTR of their target genes, thus affecting the development and prognosis of different cancers [30,31]. miRNA polymorphism could also affect diseases, for example, hereditary spastic paraplegia, hypertension, and drug resistance [32]. The miRNA-608 gene is located in the SEMA4G gene intron and belongs to the immunoglobulin family. Abnormal expression of miRNA-608 has been reported in cases of colorectal cancer [16,33], breast cancer [34], ovarian cancer [35], squamous cell carcinoma of the esophagus [36], and notochordoma [37]. Neoplastic cells usually overexpress pro-inflammatory cytokines, such as proteases, polyunsaturated fatty acid, cytokines, and chemokines. Multiple cytokines, including tumor necrosis factor (TNF)-α, interleukin (IL)-6, IL-17, IL-12, IL-23, IL-10, transforming growth factor (TGF)-β, and macrophage migration inhibitory factor, have been associated with human cancers and can promote or inhibit tumor development. miRNA-608 has been found to affect IL-6 expression levels, and IL-6 concentrations were notably higher in KD patients [38]. We aimed to determine whether there was a relationship between miRNA-608 and KD. Therefore, we conducted the present study with a large quantity of KD cases, including 532 KD cases and 623 healthy children from the Southern Chinese population, and miRNA-608 was selected as the study subject.

Our study is the first to examine the *miRNA-608* rs4919510 G>C polymorphism in KD patients. The association between KD and *miRNA-608* rs4919510 G>C polymorphism was analyzed in the present paper. We found no significant relationships between *miRNA-608* rs4919510 G>C polymorphism and KD susceptibility. The results of this experiment showed that the rates of the *miRNA-608* rs4919510 G>C polymorphism in different gender groups and age groups were not different. The major complications in KD are CAL and CAA. In the stratified analysis, we found that the *miRNA-608* rs4919510 CG/CC genotypes could increase the coronary injury risk in KD patients. The present study showed that the *miRNA-608* rs4919510 G>C polymorphism may be a biomarker for the prognosis of KD.

The current study has some limitations. First, we only analyzed the population in Southern China, which is subjected to geographical factors. We only obtained the patients’ gender, age, CAL, and CAA information and ignored the impact of family and birth history. These factors also affected the result that miRNA-608 rs4919510 G>C polymorphism confers reduced coronary injury. Second, we ignored the patient’s infection and medication history and eating habits. These factors could affect the intestinal flora. One study showed that KD patients had a significantly lower incidence of lactobacillus than disease control patients and healthy children [39], so there may be a relationship between intestinal flora and KD. Further studies will include these factors to confirm the results.

## Abbreviations

CAA: coronary artery aneurysm
CAL: coronary artery lesion
CI: confidence interval
KD: Kawasaki disease
NCAA: noncoronary artery aneurysm
NCAL: noncoronary artery lesion
OR: odds ratio
SNP: single nucleotide polymorphism
